# Improving shared decision making for lung cancer treatment by developing and validating an open-source web based patient decision aid for stage I–II non-small cell lung cancer

**DOI:** 10.3389/fdgth.2023.1303261

**Published:** 2024-03-22

**Authors:** Iva Halilaj, Anshu Ankolekar, Anouk Lenaers, Avishek Chatterjee, Cary J. G. Oberije, Lisanne Eppings, Hans J. M. Smit, Lizza E. L. Hendriks, Arthur Jochems, Relinde I. Y. Lieverse, Janita E. van Timmeren, Anke Wind, Philippe Lambin

**Affiliations:** ^1^ The D-Lab, Department of Precision Medicine, GROW-School for Oncology, Maastricht University, Maastricht, Netherlands; ^2^ Health Innovation Ventures, Maastricht, Netherlands; ^3^ Kheiron Medical Technologies, London, United Kingdom; ^4^ Rijnstate Hospital, Arnhem, Netherlands; ^5^ Department of Pulmonary Diseases, GROW School for Oncology and Reproduction, Maastricht University Medical Center, Maastricht, Netherlands; ^6^ Department of Internal Medicine, Catharina Hospital, Eindhoven, Netherlands; ^7^ Department of Radiation Oncology, Radboud University Medical Centre, Nijmegen, Netherlands

**Keywords:** shared decision-making, open-source iPDA, participative medicine, NSCLC, patient decision aid

## Abstract

The aim of this study was to develop and evaluate a proof-of-concept open-source individualized Patient Decision Aid (iPDA) with a group of patients, physicians, and computer scientists. The iPDA was developed based on the International Patient Decision Aid Standards (IPDAS). A previously published questionnaire was adapted and used to test the user-friendliness and content of the iPDA. The questionnaire contained 40 multiple-choice questions, and answers were given on a 5-point Likert Scale (1–5) ranging from “strongly disagree” to “strongly agree.” In addition to the questionnaire, semi-structured interviews were conducted with patients. We performed a descriptive analysis of the responses. The iPDA was evaluated by 28 computer scientists, 21 physicians, and 13 patients. The results demonstrate that the iPDA was found valuable by 92% (patients), 96% (computer scientists), and 86% (physicians), while the treatment information was judged useful by 92%, 96%, and 95%, respectively. Additionally, the tool was thought to be motivating for patients to actively engage in their treatment by 92%, 93%, and 91% of the above respondents groups. More multimedia components and less text were suggested by the respondents as ways to improve the tool and user interface. In conclusion, we successfully developed and tested an iPDA for patients with stage I–II Non-Small Cell Lung Cancer (NSCLC).

## 1 Introduction

Lung cancer is the leading cause of cancer deaths in Europe, with more than 410,000 people diagnosed every year (1, 2). Approximately 85% of patients with lung cancer are affected by non-small cell lung cancer (NSCLC) (3). Almost 25% of NSCLC cases are detected at an early stage, when the cancer is still manageable with radical treatment (4). There are two main effective treatment options for early-stage NSCLC: surgery and stereotactic radiotherapy (SBRT) (5). According to the Dutch guidelines for lung cancer treatment, lobectomy with lymph node dissection is the treatment of preference for patients with stage I–II NSCLC. However, evidence on the superiority of lobectomy over SBRT is lacking in literature. SBRT may be associated with a lower percentage of short-term treatment-related complications than surgery (6). Therefore, for patients with an increased surgical risk (i.e., due to lung function, cardiovascular profile, or vulnerability), SBRT can be considered a preferred treatment option. Other factors, such as the location and characteristics of the tumor, should also be taken into account in determining the treatment of choice (7, 8).

Based on the literature, it cannot be concluded whether NSCLC patients and relatives have a general preference for either surgery or SBRT. Hence, it is advised to provide patients with both treatment options and to discuss the guidelines as well as the pros and cons of both options (7).

Shared decision-making (SDM) has been reported to improve patient participation and experience based on evidence level 1 from a meta-analysis that included trials on more than 30,000 patients (9, 10). In SDM, medical professionals and patients collaborate to weigh all available data about a patient's health and choose the appropriate course of action in light of the patient's preferences and values. However, SDM is challenging because patients must consider the pros and cons of a variety of therapy alternatives while simultaneously managing the emotional toll of their illness (10). It is essential to provide patients with sound decision assistance to enhance their quality of life and the standard of medical care (11).Recent studies have shown that patients who were guided through SDM were less fearful of their treatment and felt they had a better understanding of the risks (12, 13). Studies have demonstrated that SDM boosts patients' satisfaction with their treatment choices, lowers anxiety, and improves patients' knowledge of their conditions. Patient decision aids (PDAs) have shown great potential for helping patients make informed decisions about their healthcare. Despite the advantages of SDM and PDAs, recent research has shown that a large number of patients with NSCLC encounter decisional conflict and believe they are uninformed about treatment alternatives. It has been reported that many patients with early-stage NSCLC value their involvement in treatment decision making (11). Although there are PDAs available, there is a clinical need for an open-source, individualized, patient decision aid (iPDA) specifically for patients with stage I–II NSCLC, that takes into account patients' clinical data and preferences (12, 14). Such an iPDA can empower patients to make informed decisions regarding treatment that align with their personal values. In this study, we developed a web-based iPDA for patients with early-stage NSCLC. The iPDA was designed to provide easy-to-understand information about treatment options and assist patients in making informed decisions. The aim of this paper is to explore end-users’ perspectives and assess the potential of the developed web-based iPDA to improve patient knowledge about treatment options and increase overall patient satisfaction.

## 2 Materials and methods

### 2.1 Development of the iPDA

**Figure 1:**
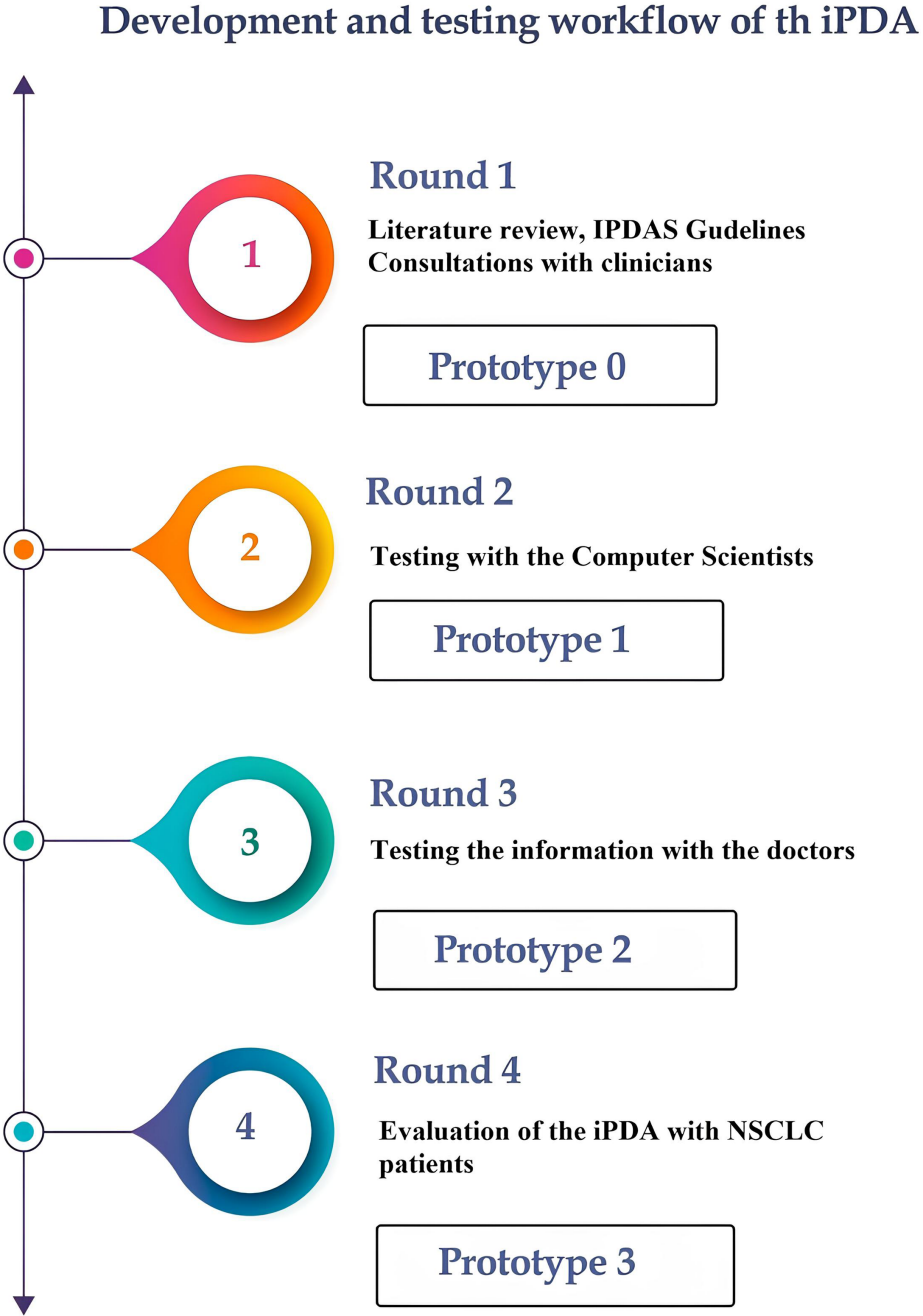
The process from the research phase until the final prototype of the iPDA.

In Figure 1, we illustrate our approach to developing the decision aid. It involved extensive consultations with healthcare professionals, particularly pulmonologists, to incorporate expert insights, alongside a meticulous literature review and alignment with Dutch national guidelines to ensure evidence-based and guideline-compliant content.

*Round 1:* The International Patient Decision Aid Standards (IPDAS) served as the guiding principles in the development of our iPDA for patients with lung cancer (15). The first iPDA version (Prototype 0) was developed in compliance with Dutch guidelines for NSCLC and after consulting with pulmonologists and radiation oncologists (16). The Internal Review Board (IRB) of Maastricht UMC and Rijnstate Hospital reviewed and approved this study, and written informed consent was obtained from all respondents. The study was registered at Home—ClinicalTrials.gov (NCT04375566: Progressive Web App About Individualized Patient Decision Aid for NSCLC Stage I–II).

*Round 2:* After the first draft was finished, the app was tested by 28 computer scientists (from the Precision Medicine Department of Maastricht University) to determine its usability and functionality. Prototype 1 was launched following this round of testing.

*Round 3:* Twenty-one physicians from multiple clinical specialties, such as radiation oncology, pulmonology, surgery, and general practice, tested the app to assess its compliance with guidelines and the reliability of the information provided. The questionnaire primarily focused on the information provided by the app, including treatment options, short-term side effects, complications, the likelihood of developing long-term complications, and frequently asked questions. The physicians assessed the accuracy of these features. The prototype 2 was online after this round of testing.

*Round 4*: The final round of our study gathered feedback from patients with NSCLC regarding the informational and decision-making needs related to the iPDA. This round of the study consisted of two sub-phases: semi-structured interviews and questionnaires (see “Supplementary Materials”).

#### 2.1.1 Patients inclusion criteria

A small group of patients (see “Supplemental Materials” section, METC (Medical Ethics Review Committee (in Dutch—Medisc-ethische toetsingscommissie) approval request, non-WMO (Research subject to the WMO—plichtig onderzoek)) who had been treated for early stage NSCLC were invited to participate in this study. The recruited patients had received radiotherapy or surgery at least 6 months prior and were currently free of disease with no progression. This patient group was chosen because their burden would be lower than that of other patients with progressing diseases. Additionally, patients needed to be at least 18 years old to participate. The treating pulmonologist asked the patients if they were willing to participate in the pilot study, and when they had read the patient information sheet, the researcher scheduled an appointment, and written informed consent was obtained before study-related procedures. They were then given a questionnaire to complete. The study had minimal impact, with no medical examinations or procedures involved. There were no associated risks, and the burden was low, consisting of a single semi-structured interview and a 40-item questionnaire. The patients had the opportunity to review the entire tool (by following the instruction from the researcher) with the support and presence of their partners or family members. Following this review, they proceeded to assess the iPDA, having received information about stereotactic radiotherapy and surgery.

#### 2.1.2 Semi-structured interviews

During the first phase, semi-structured interviews were conducted with thirteen patients, which were audio-recorded and transcribed as shown in Table 1. The questions focused on the patients' role in the decision-making process for their treatment during the initial diagnosis of early-stage NSCLC. Patients were asked regarding their engagement in the decision-making process, the role of family and friends, the hardest part of making decisions, information that was significant to them, and whether they had researched additional information. Patients were also questioned about how they preferred to learn about various treatment options and what aspects of the treatment selection process they would change.

**Table 1:** Steps taken for interview interpretations.

Step	Action
1	Read through the interview transcripts and make notes
2	Go through the notes and list the different types of information found
3	Read through the list and categorize each item
4	Repeat the first three stages again for each interview transcript
5	Collect all of the categories or themes and examine each in detail and consider it's fit and its relevance
6	Categorize all data (all transcripts together) into minor and major categories/themes
7	Review all categories and ascertain whether some categories can be merged or sub-categorized
8	Return to original transcripts and ensure that all the information has been categorized

#### 2.1.3 Questionnaire

The 40-item questionnaire, based on elements of the validated System Usability Scale and a study-specific questionnaire (17, 18), assessed the performance, potential, and value of the application. Comprehensibility, usability, and the value of the information presented were all considered in the responses about satisfaction with the iPDA. Additionally, the effectiveness of the iPDA in aiding decision-making was also evaluated. The answers were given on a 5-point Likert scale that ranged from “strongly disagree” to “strongly agree”. The interviews and questionnaire focused on various themes related to the iPDA, and each interview lasted between 20 and 30 min. The final version (Prototype 3) of the iPDA was released following feedback from all participants.

### 2.2 Interview analysis

To analyze the interview transcripts, we used a qualitative content analysis approach (Table 1), which involved reading through the transcripts, categorizing the information, and examining each category's fit and relevance (19, 20). We also adapted a previously published questionnaire for the usability survey, which contained 40 multiple-choice questions and three open-ended questions. The questions of the questionnaire were selected from different validated questionnaires (18, 21). Based on the feedback we received from the respondents, we revised the prototypes of the iPDA over successive rounds to make them more user-friendly and effective in assisting patients in making informed treatment decisions.

The selected questions focus on the patients' role in the decision making process for their treatment. Patients were asked regarding their engagement in the decision-making process, the role of family and friends in treatment and decision-making, significant factors in decision-making, whether they thought information regarding treatment was lacking, and if they had searched for additional information.

### 2.3 Questionnaire analyses

The survey responses were analyzed by clustering the questions into four main groups based on their content, as illustrated in Table 2. This was done initially by the first author, and checked by the last author to check the inter-rater reliability. Descriptive analyses were performed to interpret the results, which involved percentage and frequency-based calculations. These analyses were conducted using the statistical software SPSS (22).

**Table 2:** Questions categorized into 4 groups.

Questions clusters	Questions from the questionnaire
a. Usability and design	In general the tool is easy to use.
	Navigating in the Patient Decision Aid is easy.
	The content of the tool is clear and easy to follow.
	This tool is nicely designed.
	The tool takes too much time to finish.
b. Information quality and clarity	The written information of the tool is clear.
	The information about the side effects is clear.
	The tool clearly shows the advantages and disadvantages of lung surgery.
	The tool clearly shows the advantages and disadvantages of lung radiotherapy.
	The tool gives enough details about the treatments to make a decision.
c. Impact and effectiveness	The tool helps patients to see what is important for them in a treatment.
	I believe this Patient Decision Aid can motivate patients to participate in their treatment.
	I would recommend this tool to patients.
	I believe this tool will help patients learn more about treatment options.
	I believe this tool will help patients to make an informed decision.
d. Relevance and usefulness	The written information about the treatments is useful.
	The information about the side effects is useful.
	I believe the tool is a useful Patient Decision Aid.
	The information about the different treatments was comforting.

### 2.4 Technical development of the iPDA prototypes

JavaScript, HTML5, CSS3, Ajax, jQuery, and Bootstrap were used in web development programming languages to provide a user-friendly interface that incorporated animations and movies to help people comprehend the many treatment alternatives. The tool's interoperability with patients was also achieved using the Python-based Django framework. The iPDA is designed to walk patients through each phase, making sure they are fully informed and able to make an informed decision about their care.

## 3 Results

### 3.1 User interface of the iPDA-final prototype

**Figure 2:**
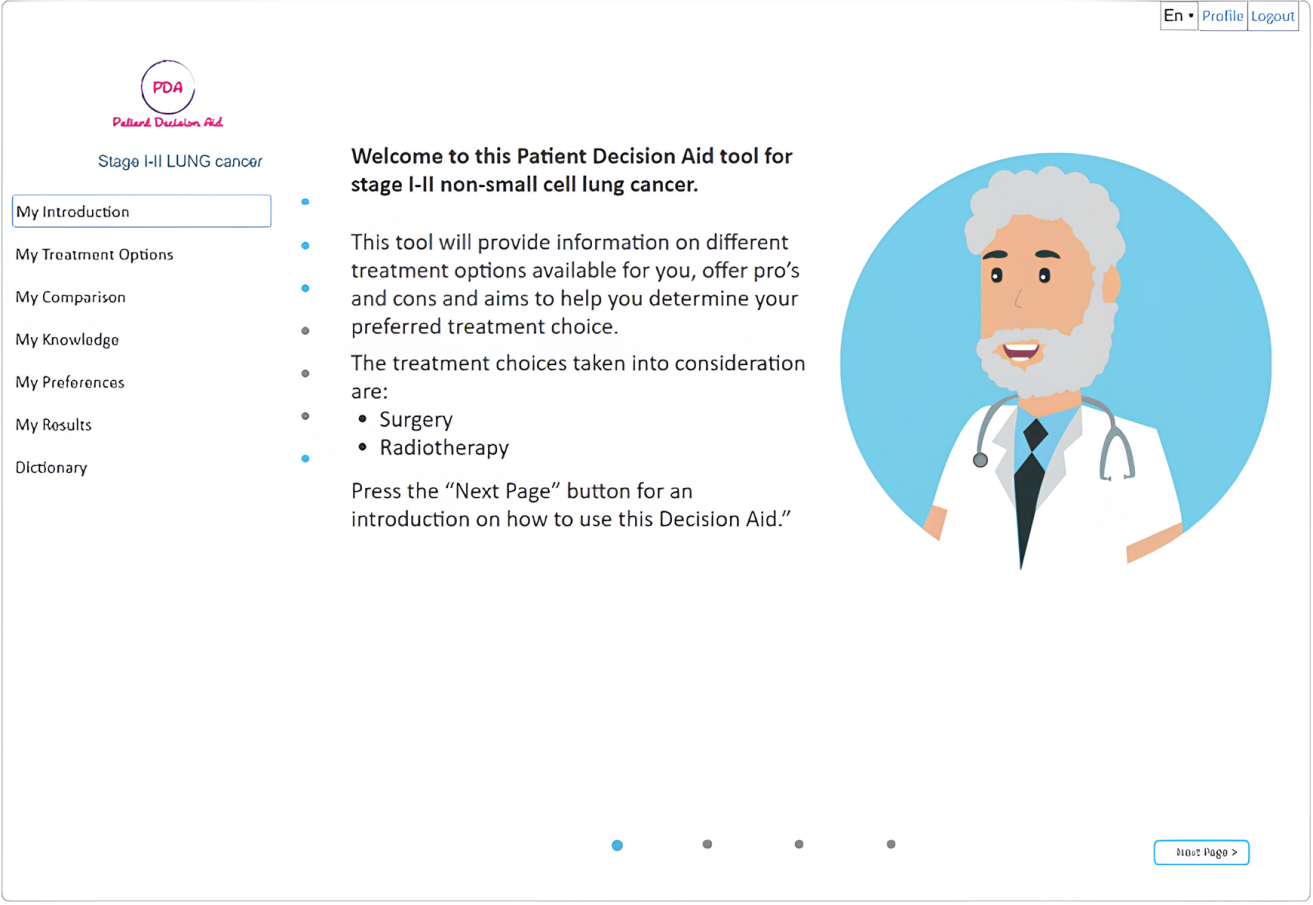
**S**hows the final web version of the iPDA.

A web-based version of the open-source (https://github.com/ivahalilaj1/iPDA.git) iPDA tool has been developed and can be accessed at iPDA Profile. A screenshot of the tool is presented in Figure 2. The iPDA provides patients with a complete overview of their treatment options and encourages them to be actively involved in the decision-making process. By doing so, the application not only saves time for healthcare professionals but also improves patient-doctor communication.

**Figure 3:**
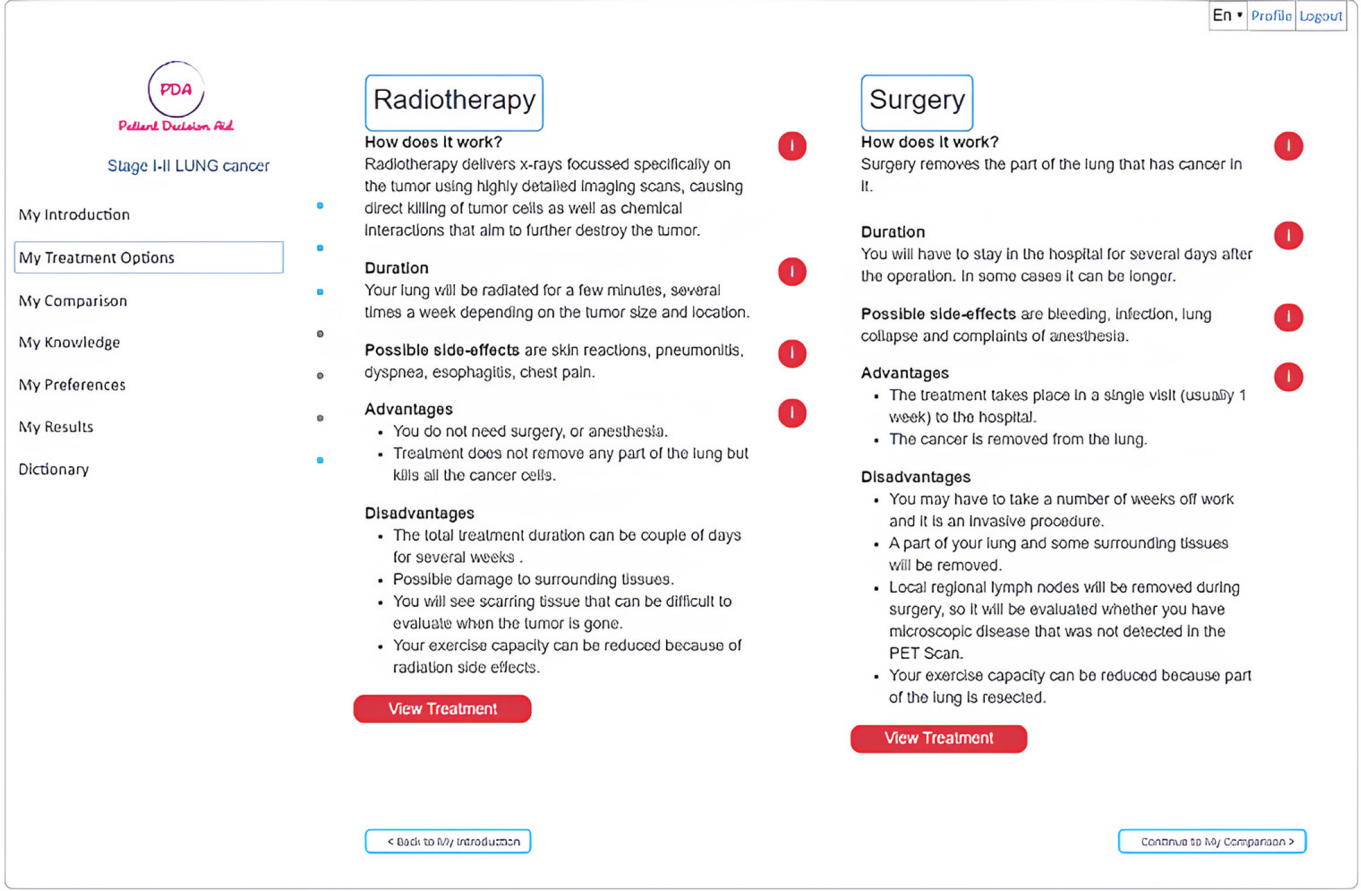
The treatment options available.

**Figure 4:**
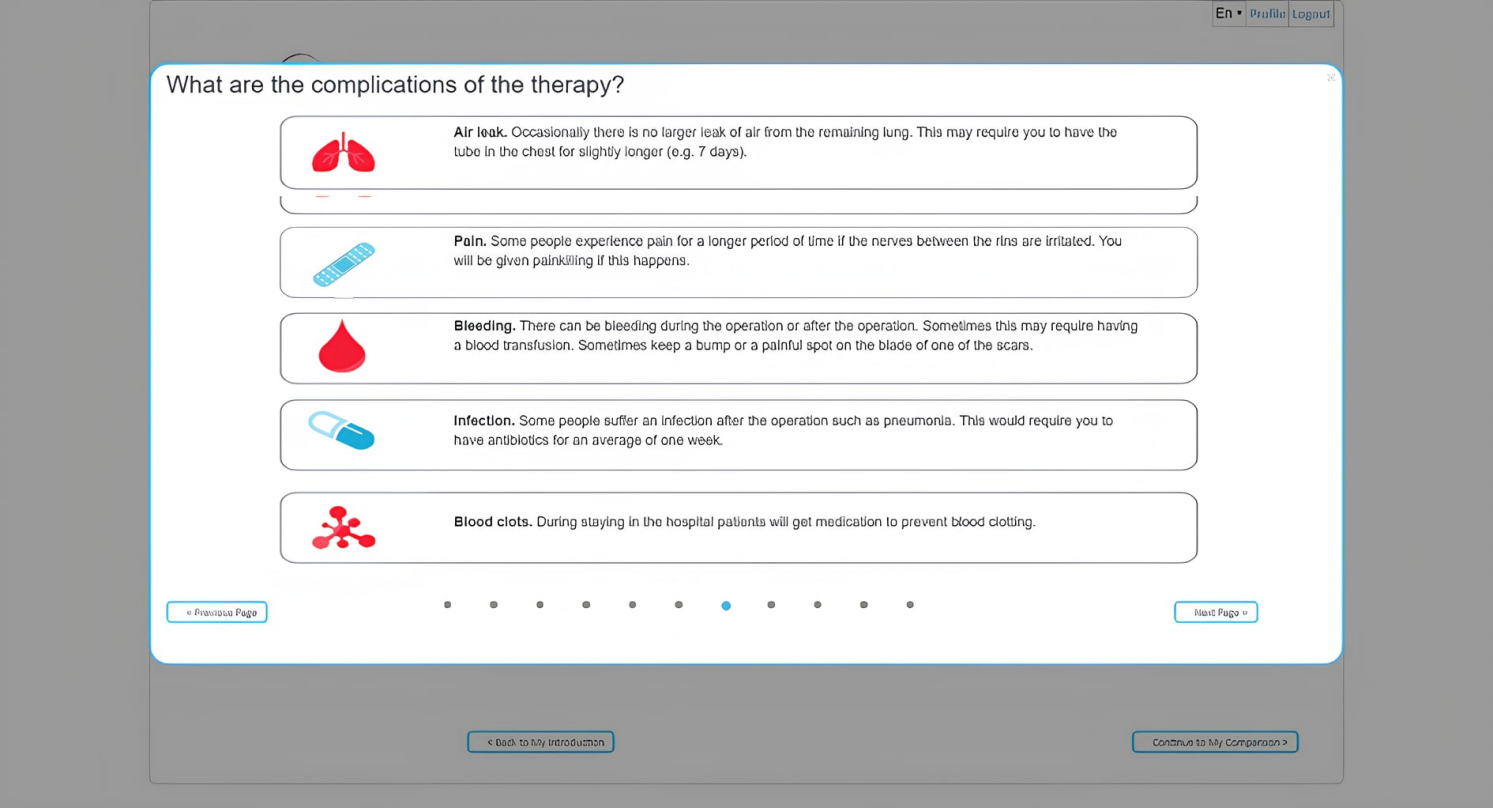
Example of a way of showing the side effects of one of the treatment options.

The homepage of the decision aid for patients with lung cancer provides general information about lung cancer as well as information about various treatment types through the use of animations, interviews with specialists, and bullet points, allowing users to receive information at different levels of detail. The iPDA is designed to guide the patient through the tool without the option to skip information that has not been previously viewed (as shown in Figure 2). The tool provides general information about lung cancer and detailed information about different treatment types using a variety of media, including animations, interviews with specialists, and bullet points (as shown in Figures 3, 4).

Different pieces of information, as shown in Figure 3, are presented using bullet points combined with video. Users can review all relevant material about the treatment options: lobectomy (or other surgical procedure for lung cancer), radiotherapy, and perioperative chemotherapy. The iPDA enables users to compare the different treatment options side by side with their respective 5-year progression-free survival, 10-year overall survival, and the probability of facing adverse events, such as radiation pneumonitis, pneumonia, empyema, air leak, and lung collapse, based on estimates for the whole treatment population.

Figure 4 demonstrates how one of the treatment options' side effects can be presented. The next step in the tool involves a knowledge test to verify that the user has received and correctly interpreted all the information. The results of this knowledge check can be discussed with a clinical professional to further explain misunderstood information. This is followed by a section in which the user rates the expected impact of an aspect of treatment (hospitalization, recovery time) or a side effect (chest pain, skin reactions, infections, reactions to anesthesia) on their quality of life using a 3-point Likert scale (1-not a Problem; 2-minor Problem; 3-Major Problem). The results of these questions are presented to the user per treatment modality using a color scheme where red indicates that the user is negatively impacted by a certain aspect of the treatment and green indicates that they are not.

### 3.2 Interview results

A total of 13 patients have been interviewed. The questions asked during the interview and a summary of the answers given are displayed in Table 3 and in the following paragraph. Eight patients indicated that they felt involved in the decision-making process regarding their treatment. For two of these patients, surgery was preferred because of the localization of the tumor. They were clearly informed about this by their physician. Of the remaining five patients, two did not feel engaged, while three stated that their participation in the decision-making was questionable, as surgery was the only option provided and it was not clear to them whether there was an alternative. Patients that did participate in decision-making reported various factors that were relevant in this process.

**Table 3:** Interview answers from the patients.

Interview questions	Answers	
Did you feel involved in the decision-making process?	• Yes (*n* = 8) • Questionable (*n* = 3) • No (*n* = 2)	
Which factors played a role in decision-making?	• Physician's opinion (*n* = 5) • Potential side effects/risks (*n* = 3) • Duration of treatment (*n* = 2) • Quality of life (*n* = 1) • Their own and others’ experiences with radiation and surgery (*n* = 1) • Recovery period (*n* = 1) • Confirmation of complete tumor removal (*n* = 1)	
Did you involve others in the decision-making process?	• Yes (*n* = 10) • No (*n* = 3)	
Who was involved during your treatment?	• Partner (*n* = 11) • Other relatives (*n* = 7) • Friends (*n* = 2) • Information on alternative treatment options (*n* = 3) • Information on possible long-term complications/symptoms after surgery (*n* = 2) • Expertise of different hospitals/surgeons (*n* = 1) • Anatomical information (*n* = 1) • Long term effects, such as pain (*n* = 1)	
What information was missing regarding treatment?		
Did you search for additional information?	**Yes/No?**	**What kind of information?**
	Yes (*n* = 8)	• Website of the patient association • Book on how to deal with cancer • Online information on different types of lung cancer • Online information on treatment options • Results of surgery in different hospitals • Website recommended by his physician + online information he found himself • Homeopath • Mortality rate
	No (*n* = 5)	**Explanation**
		• Fear, feeling depressed (*n* = 1) • All questions had been answered already (*n* = 3)

The most frequently mentioned aspects include their physician's opinion regarding the best treatment option and potential side effects and risks of treatment. Other factors that played a role were the duration of treatment, quality of life, patients' own and others' experiences with treatment, recovery period, and confirmation of complete tumor removal. The majority (92%) of patients stated that besides themselves, others (partners, family, and/or friends) were involved in both decision-making and treatment. Several patients (61%) that did not feel engaged, indicated that certain information on treatment was lacking, such as information about alternatives, possible long-term complications (i.e., pain), and anatomical information related to surgery. One of the patients that did feel informed, mentioned that he was missing information on the expertise of different hospitals and surgeons. Eight patients searched for additional information before making a decision. Most of them provided themselves with information found on the internet, i.e., on different types of lung cancer, mortality rates, treatment options, and the performance of different hospitals. Two patients mentioned that they did not feel like the information they had read was applicable to them, since they didn't recognize themselves in the patient stories. Of the five patients who did not search for information, three of them declared that their questions had already been answered by their physicians and/or nurses. One patient didn't search the internet because of negative experiences with this in a previous disease process.

The first column shows the interview questions. The other two columns show a summary of answers and the number (*n*) of patients that provided that answer.

### 3.3 iPDA questionnaire results

#### 3.3.1 Usability and design

Regarding general usability, the majority of respondents found the tool to be user-friendly. Among physicians, 81% agreed with this statement, while 85% of patients and 96% of computer scientists shared the same opinion. About navigating within the iPDA, the majority of respondents (91% of physicians, 78% of patients, and 82% of computer scientists) expressed that it was easy.

**Figure 5:**
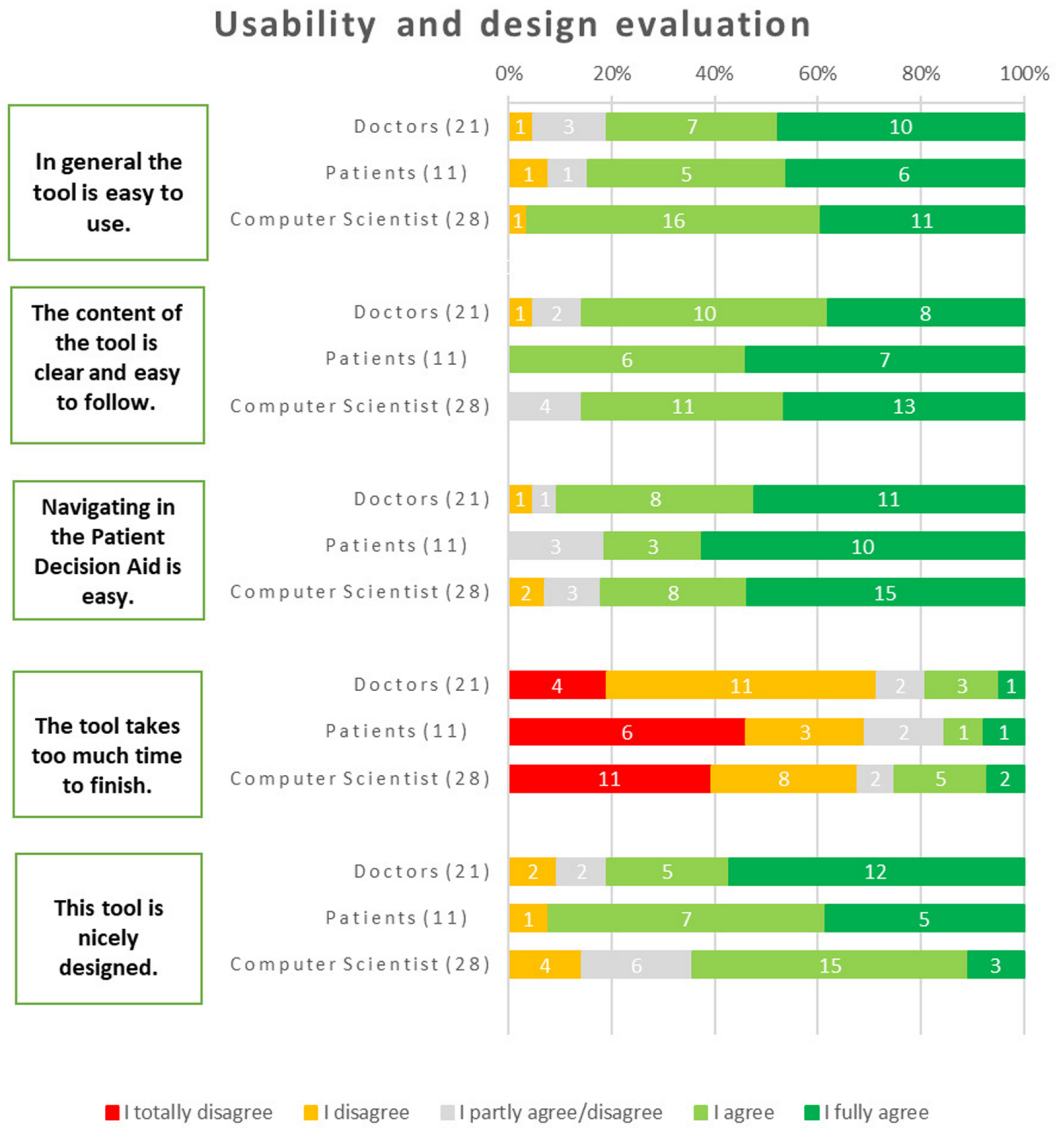
Results about user friendliness and design.

Regarding whether it took too much time to use the tool, 68% of computer scientists disagreed with the statement (25% agreed, and 7% remained neutral). Similarly, 71% of physicians expressed a similar opinion (with 10% remaining neutral and 19% agreeing). Likewise, 69% of patients shared the perspective that the tool did not take too much time to finish (with 15% neutral and 15% in agreement). The design of the tool received positive feedback from the majority of respondents: 81% of doctors, 64% of computer scientists, and most critically, 92% of patients expressed their liking for the design of the tool (Figure 5).

The primary criticisms were as follows, in terms of what they wanted the iPDA to change:

• “The Results” tab might be made into a dashboard to make navigating easier.
• The quiz's “slide” transitions should become more automated, which will improve the user experience.
• Upon completion of the test, permitting free navigation will allow users to assess the information whenever they are ready.

#### 3.3.2 Information quality and clarity

For the clarity of the videos about the treatments, the majority of physicians (81%) agreed that they were clear and easily understandable. The questions regarding the tool's ability to clearly present the advantages and disadvantages of lung surgery and lung radiotherapy were exclusively directed towards physicians, considering their clinical expertise. The responses from physicians indicated a high level of agreement, with 95% agreeing that the tool effectively conveyed the information for lung surgery and 91% agreeing in the case of lung radiotherapy. These results emphasize the tool's efficacy in providing comprehensive and clear information about the advantages and disadvantages of these treatment options, as perceived by medical professionals. All patients unanimously agreed that the written information provided by the tool is clear and easily comprehensible. The clarity of the written information within the tool received positive feedback across all respondent groups. Specifically, 92% of patients, 95% of physicians, and 97% of computer scientists agreed that the written information was clear and easily understandable.

**Figure 6:**
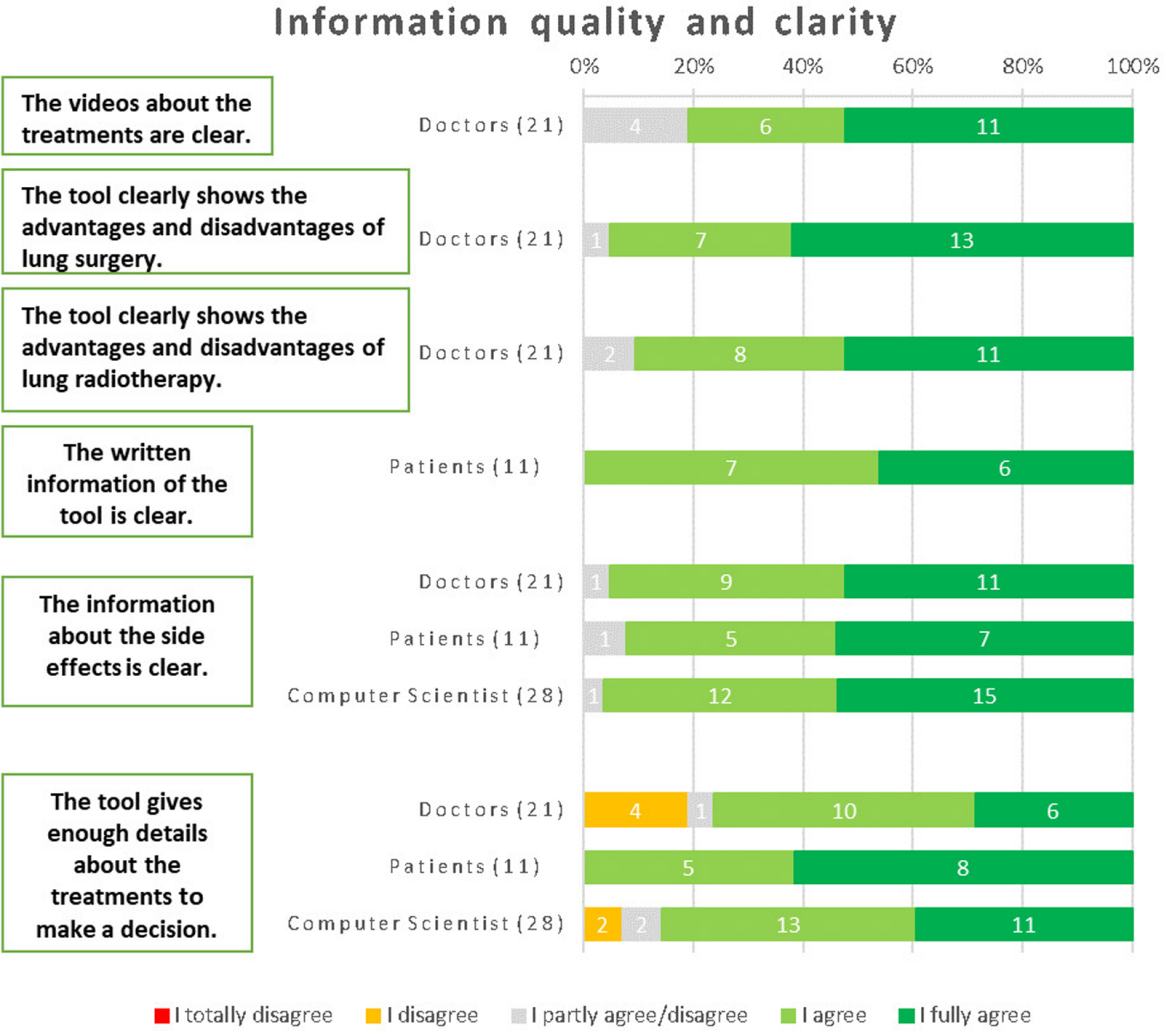
Results from the respondents about information quality (physicians) and clarity (patients).

In terms of providing sufficient details about the treatments to make informed decisions, all patients unanimously agreed that the tool fulfilled this requirement. Among physicians, 76% agreed with this statement, 19% disagreed, and 5% remained neutral. Similarly, 77% of computer scientists also agreed that the tool provided enough details about the treatments (Figure 6). The main recommendations to improve the information were: making videos longer and more informative will help them be optimized; using more multimedia components and less text; and having a button for urgent assistance that initiates a discussion with a professional online.

#### 3.3.3 Impact and effectiveness

**Figure 7:**
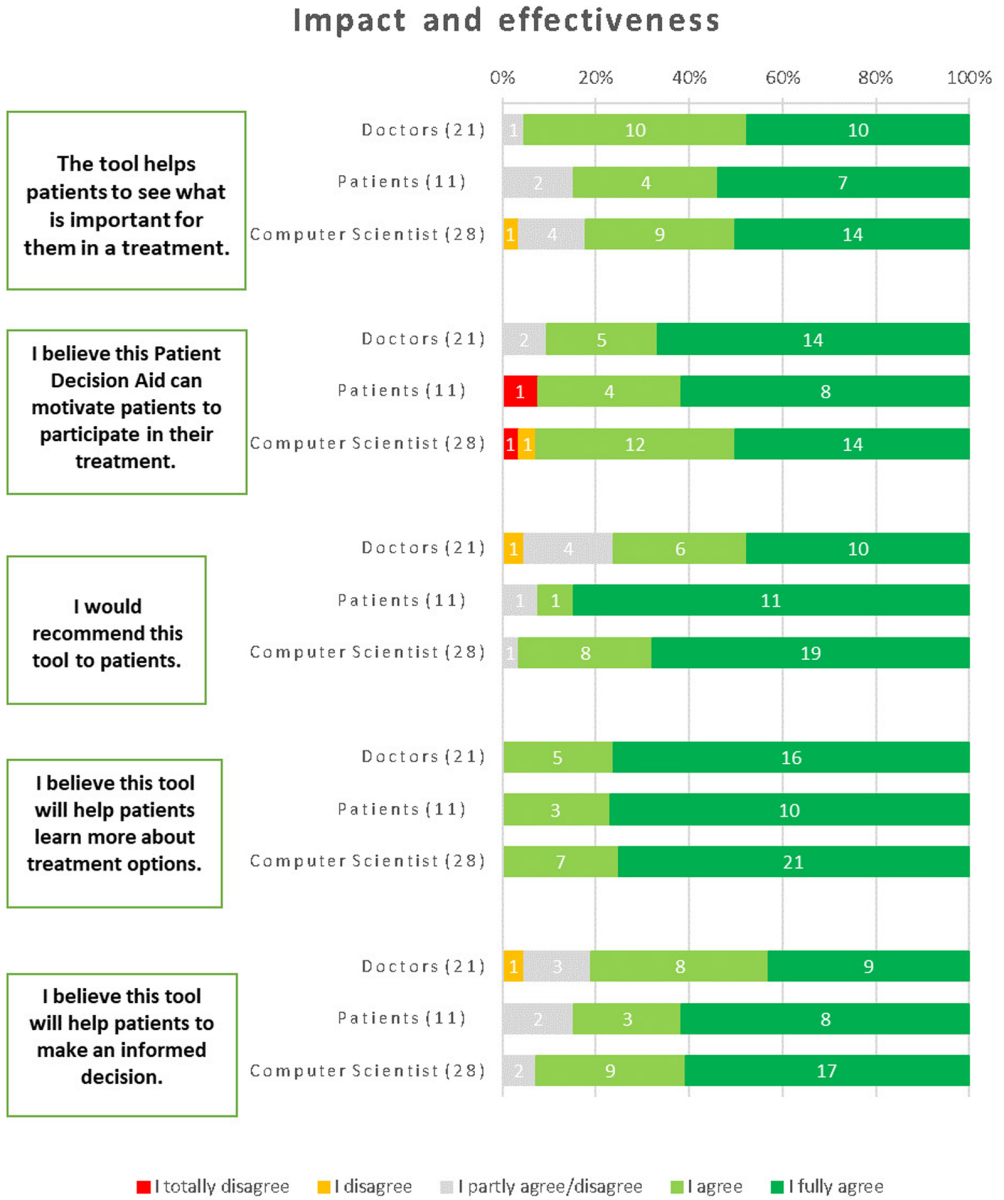
Results about the impact and effectiveness of the iPDA.

The majority of respondents recognized the tool's ability to assist patients in identifying what is important to them when considering a treatment. Specifically, 85% of patients agreed, 94% of physicians approved, and 82% of computer scientists shared the same sentiment. The respondents demonstrated a strong belief that the iPDA has the potential to motivate patients to actively engage in their treatment. This sentiment was shared by 93% of computer scientists, 92% of patients, and 91% of physicians. A significant majority of respondents expressed their willingness to recommend the tool to patients. Among physicians, 76% agreed with this statement, while 92% of patients and 97% of computer scientists also shared the same opinion. There was unanimous agreement among computer scientists, patients, and physicians regarding the tool's ability to help patients gain a deeper understanding of treatment options. The respondents expressed a strong belief that the tool aids patients in making informed decisions. Among physicians, 81% confirmed this viewpoint, while 77% of patients and 93% of computer scientists agreed as well (Figure 7).

Recommendations to improve the toll concerned adding personalized predictions concerning genetic, hematologic, and clinical parameters. Additionally, different diagnostic choices, cost information, and treatment notes could be incorporated to give a comprehensive overview.

#### 3.3.4 Relevance and usefulness

**Figure 8:**
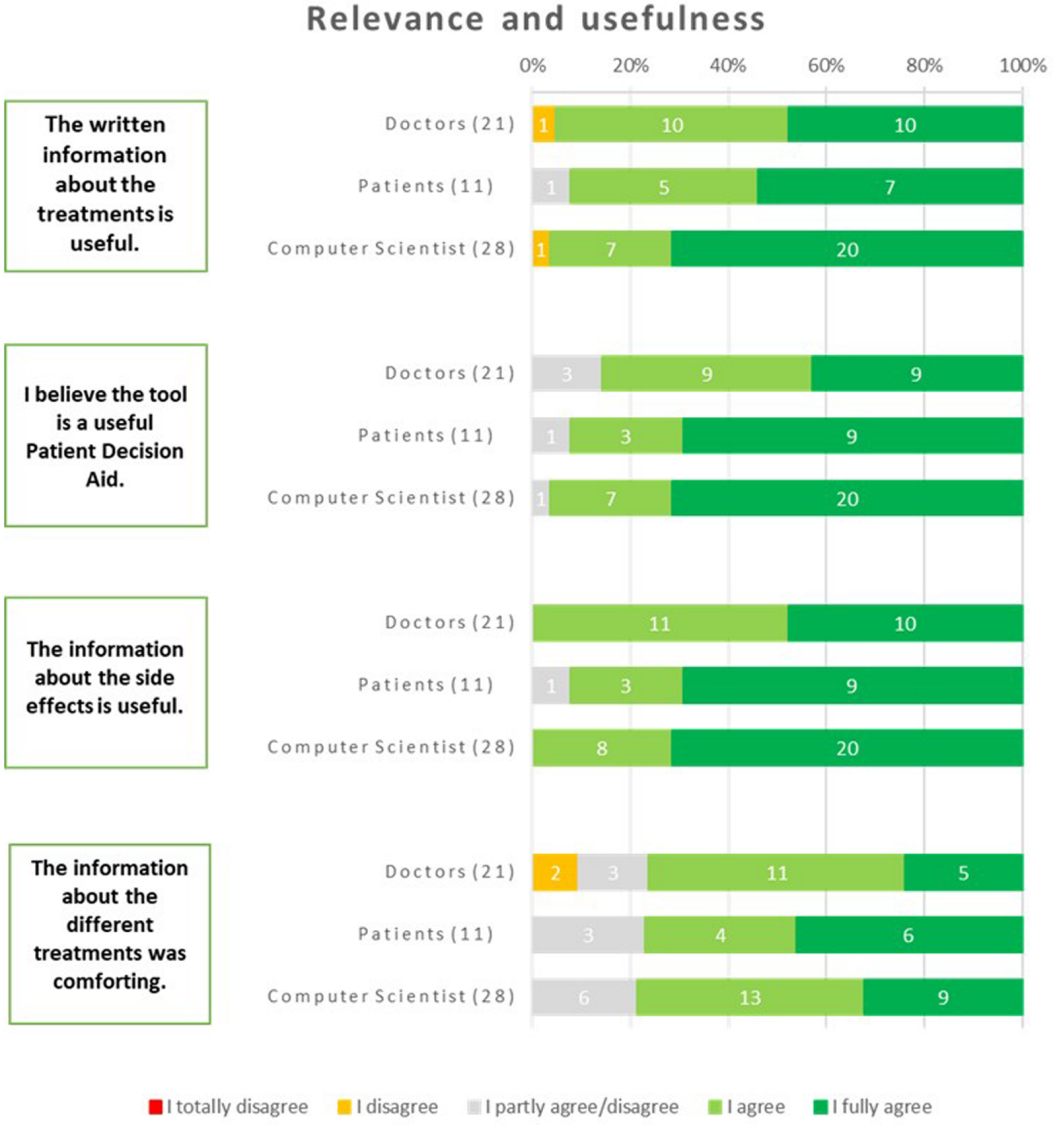
Results about the relevance and usefulness of the iPDA.

The respondents regarded the tool as a valuable iPDA. Among patients, 92% expressed this belief, while 96% of computer scientists and 86% of physicians shared the same opinion. The respondents widely acknowledged the written information about the treatments as useful. This consensus was evident among physicians, with 95% expressing agreement, and among patients, with 92% expressing the same sentiment. Additionally, computer scientists, with a majority of 96%, also agreed with this statement. The usefulness of the information about the side effects received unanimous agreement from all physicians and computer scientists. Additionally, 92% of patients also found this information useful. The information about the different treatments was found to be comforting by a significant portion of the respondents. Among patients, 77% expressed this sentiment, while 71% of physicians and 76% of computer scientists shared the same viewpoint (Figure 8).

Suggestions from the respondents were to incorporate comprehensive data, quantitative comparisons, and decision-making support so the users would have access to valuable information while making informed healthcare choices.

The overall evaluation of the iPDA by doctors yielded a score of 7.6 out of 10, indicating a generally positive assessment. Patients, on the other hand, provided a higher rating, with an overall evaluation of 8.6. Similarly, computer scientists evaluated the iPDA with an overall score of 7.8. These ratings reflect the overall satisfaction and perception of the iPDA across different respondent groups.

## 4 Discussion

This paper presents the development and qualitative evaluation of an open-source web-based iPDA for lung cancer in its early stages. The iPDA was tailored to patients’ treatment preferences and potential side effects. The results of our study, which looked at how end users perceived the web-based iPDA, show that the tool was successful in serving its intended function. Our goal was to determine whether the iPDA was successful in delivering clear information about available treatments and assisting patients in making informed decisions. We can confidently state that the iPDA substantially achieved its goals based on the feedback and responses we received from medical professionals, patients, and computer scientists.

• Participants praised the iPDA's simplicity of use and how user-friendly it was to use. Medical professionals were positive about its capacity to concisely and clearly express benefits and drawbacks when providing information about available treatment alternatives.
• Patients, physicians, and computer scientists all viewed the iPDA favorably as a useful tool for assisting patients in identifying critical aspects of treatment decisions. Patients were successfully encouraged to actively participate in their decision-making process and obtain a greater awareness of their options.
• All respondents thought the iPDA was valuable, proving its applicability and potential value in clinical situations. Patient needs and concerns were effectively met by the information, which was particularly helpful and reassuring regarding side effects and long-term consequences.

This study showed that this initial version of the lung iPDA serves as a robust and easily accessible tool that effectively delivers information and helps decision-making processes.

The conducted qualitative analyses through interviews to gain in-depth insights into the patients' perspectives and their experiences with the iPDA app. This qualitative approach provided valuable qualitative data complementing the quantitative questionnaire analysis. These findings align with other studies focused on various cancer types, such as prostate cancer, laryngeal cancer, breast cancer, and more (23–29). Consistency across different studies and cancer types strengthens the evidence supporting the positive impact of PDAs in healthcare. This study used a mixed-methods approach, followed by Dutch guidelines for lung cancer treatment, and adhered to the IPDAS Evidence Update 2.0 recommendations (15, 17).

As this study only covered the early stages of the development and validation of the iPDA, further development is needed to have a fully validated iPDA. This discussion focuses on the steps, limitations, and challenges involved in the ongoing development of the tool:

Firstly, incorporating personalized predictions (considering genetic, hematologic, and clinical parameters, along with diagnostic choices) to maximize accuracy and relevance. In addition, incorporating quantitative comparisons such as survival percentages, success rates, and risk assessments enables users to have a clearer understanding of the potential outcomes associated with each treatment choice.

One approach would be to enhance the personalization of the iPDA by incorporating data from prediction models accessible at https://ai4cancer.herokuapp.com/ (30). This integration would enable the provision of personalized information on survival rates and potential side effects, thereby fostering a more individualized approach to shared decision-making. However, such models are mostly designed for clinicians and may contain information unknown to patients, therefore requiring the intervention of nurses or physicians to ensure understanding and clarity.

Secondly, the tool's information about treatment options may vary across countries. As a result, national regulations should be followed and specific updates should be made. Additionally, language barriers could prevent the tool from being used internationally.

Thirdly, when generalizing the results, it is important to consider the study's sample size and demography. It may be beneficial to engage more patients to validate and expand on these results. Additionally, the app was only tested retrospectively with patients who had already undergone curative intent treatment, so evaluating it proactively with patients who still have to be informed and have to decide on treatment would be a useful way to determine the actual impact of using the iPDA on treatment decisions and patient outcomes over time.

Another possible limitation of this study is that it only included patients from two Dutch hospitals, limiting the generalizability of the findings to a broader population, especially regarding physician-patient communication. Another limitation is that one patient only reviewed the iPDA and filled in the questionnaire, but chose not to be interviewed, and another patient who was interviewed did not complete the follow-up questionnaire. Moreover, the limited number of patients can be attributed to recruitment challenges, notably exacerbated by the obstacles presented by the COVID-19 pandemic, which substantially hampered our capacity to include a more extensive patient cohort.

Finally, the age and computer literacy of the patient population also present a challenge, as some patients may be dependent on others for the use of these tools.

Clinical integration is the next step after fully validating the iPDA (28, 31, 32). This can face challenges due to clinicians' potential lack of faith in the tool, inadequate training on its implementation, and the multidisciplinary nature of the iPDA. Overcoming these obstacles requires addressing concerns, providing comprehensive training, and carefully managing the tool's multidisciplinary aspects (13, 33). More precisely, implementing an iPDA in a clinical setting entails personalizing the tool, conducting pilot studies, involving stakeholders, incorporating it into workflows, reviewing the results, and continuously enhancing its use. By doing so, successful implementation in clinical practice can be achieved, leading to enhanced decision-making processes, more patient understanding, and improved patient outcomes.

The use of PDAs has demonstrated promising outcomes in improving patient knowledge and decision-making, despite facing certain challenges and limitations. These findings align with the positive feedback received from our respondents regarding the iPDA's usability, design, information quality, clarity, impact, effectiveness, relevance, and usefulness. This highlights the significance of continuously developing and integrating such tools in healthcare.

While our study focused on the usability and acceptability of the iPDA tool, we acknowledge the importance of assessing its impact on decision-making outcomes. Conducting a study with a control and intervention cohort to evaluate the tool's influence on patient decisions is indeed a valuable direction for future research.

The iPDA must be kept updated with the most recent discoveries and therapeutic advances. For example, new treatment techniques like perioperative immunotherapy (for surgical patients, not SBRT) and adjuvant targeted therapy for certain patient populations have been introduced, which has led to an ongoing evolution in lung cancer treatment (34–36). For this reason, developers of iPDA's and physicians must constantly work together. Setting up a governance structure on a national basis (as treatment recommendations should be the same across a country) might be helpful to streamline this procedure. Maintaining the iPDA and keeping it up to date with new treatment options (targeted therapy and immunotherapy), gathering and examining data regarding its efficacy, and disseminating the results to physicians and the larger medical community in an effort to increase awareness would all fall under the purview of this framework (37, 38). Future research and development efforts will focus on enhancing the ethical aspects of the iPDA. This includes the integration of real-time expert feedback and the refinement of how survival rate information is presented.

## 5 Conclusions

Although there is work to be done to implement this tool in a clinical setting, which requires, for example, incorporating predictive models for different treatment options and adapting the tool to the specific clinical setting where it will be implemented, in this study, we made some important first steps towards the development of an individualized iPDA; the development spanned over 4 rounds, and the prototype was evaluated multiple times with different end-users, leading to a positive judgment.

## Data Availability

The raw data supporting the conclusions of this article will be made available by the authors, without undue reservation.
