# Supplementary material for: Improving shared decision making for lung cancer treatment by developing and validating an open-source web based patient decision aid for stage I–II non-small cell lung cancer
Source: Front Digit Health. 2024 Mar 22;5:1303261. doi: 10.3389/fdgth.2023.1303261 (PMC10995236; doi:10.3389/fdgth.2023.1303261)
Supplement: Supplementary file 1 [file Datasheet1.pdf]

1. Questionnaire to assess effectiveness of the lung patient decision aid

	Strongly disagree	Disagree	Neutral	Agree	Strongly agree
1. Patients will not need help from others to go through the tool.	☐	☐	☐	☐	☐
2. The instructions in the patient decision aid are clear.	☐	☐	☐	☐	☐
3. The aim to use this patient decision aid is clear.	☐	☐	☐	☐	☐
4. This tool is good for giving information.	☐	☐	☐	☐	☐
5. This tool is nicely designed.	☐	☐	☐	☐	☐
6. The written information of the tool is clear.	☐	☐	☐	☐	☐
7. The videos about the treatments are clear.	☐	☐	☐	☐	☐
8. The written information about the	☐	☐	☐	☐	☐

treatments is useful.					
9. The videos about the treatments are useful.	☐	☐	☐	☐	☐
10. The information about the side effects is clear.	☐	☐	☐	☐	☐
11. The information about the side effects is useful.	☐	☐	☐	☐	☐
12. The tool clearly shows the advantages and disadvantages of lung surgery.	☐	☐	☐	☐	☐
13. The tool clearly shows the advantages and disadvantages of lung radiotherapy.	☐	☐	☐	☐	☐
14. The tool helps patients to make a decision for a treatment.	☐	☐	☐	☐	☐
15. The tool helps patients to see what is important for them in a treatment.	☐	☐	☐	☐	☐
16. I would recommend this tool to every patient with lung cancer.	☐	☐	☐	☐	☐
17. The tool takes too much time to finish.	☐	☐	☐	☐	☐
18. The information about the different	☐	☐	☐	☐	☐

Confidential

treatments was comforting.					
19. In general, I am satisfied with this Patient Decision Aid.	◻	◻	◻	◻	◻
20. I believe Patient Decision Aids can improve the healthcare quality.	◻	◻	◻	◻	◻
21. I believe this Patient Decision Aid can motivate patients to participate in their treatment.	◻	◻	◻	◻	◻
22. The tool works well.	◻	◻	◻	◻	◻
23. In general the tool is easy to use.	◻	◻	◻	◻	◻
24. It is easy to learn how to use the Patient Decision Aid.	◻	◻	◻	◻	◻
25. Navigating in the Patient Decision Aid is easy.	◻	◻	◻	◻	◻
26. It is clear how the tool should be used.	◻	◻	◻	◻	◻
27. I believe the tool is a useful Patient Decision Aid.	◻	◻	◻	◻	◻
28. I believe this tool will help patients learn more about treatment options.	◻	◻	◻	◻	◻

29. I believe this tool will help patients to make an informed decision.	☐	☐	☐	☐	☐
30. I would recommend this tool to patients.	☐	☐	☐	☐	☐
31. I believe every patient in my hospital should be able to use a decision aid when possible.	☐	☐	☐	☐	☐
32. The tool gives enough details about the treatments to make a decision.	☐	☐	☐	☐	☐
33. The content of the tool is clear and easy to follow.	☐	☐	☐	☐	☐
34. The information presented in the tool is correct.	☐	☐	☐	☐	☐
35. The Patient Decision Aid will make a doctors visit take longer.	☐	☐	☐	☐	☐
36. I think this tool is also relevant and helpful for patient's family members.	☐	☐	☐	☐	☐
37. I believe this tool will make the communication between the doctor and the patient easier.	☐	☐	☐	☐	☐
38. I believe that a mobile version of the	☐	☐	☐	☐	☐

tool will be easier to use.	
39. The tool is visually attractive.	☐ ☐ ☐ ☐ ☐
40. The time needed for following this tool was:	
41. What is you general evaluation for the tool (1-10)?	
42. Which aspects of the Decision Aid did you like?	
43. What do you want to change in the iPDA?	
44. Do you think something is missing in the tool?	
45. Do you have any additional remarks?	

2. Patient information

Patiënten informatie

<u>Titel van de studie</u>

Evaluatie van een beslishulp voor gezamenlijke besluitvorming met betrekking tot behandeling van vroeg stadium niet-kleincellig longkanker.

Geachte heer/mevrouw,

Wij verzoeken u vriendelijk dit informatieformulier door te lezen.

lnleiding

Op dit moment zijn er soms meerdere mogelijkheden om gelokaliseerde longkanker te behandelen. Hierdoor is het mogelijk om patiënten meer te betrekken in het maken van een keuze voor een behandeling. Om ervoor te zorgen dat patiënten alle informatie krijgen om de juiste beslissing te nemen hebben wij een beslishulp ontwikkeld.

Een beslishulp helpt de artsen en patiënten bij het kiezen van een optimale behandeling. Voordelen van een beslishulp zijn:

- duidelijke en gerichte informatievoorziening,
- meten en weten van individuele waarden en verwachtingen van patiënten ten aanzien van de behandeling,
- artsen krijgen beter inzicht in de waarden en verwachtingen van patiënten,
- patiënten zijn meer betrokken in de gezamenlijke besluitvorming met de arts voor een optimale behandeling,
- betere kwaliteit van de medische zorg.

De beslishulp die is ontwikkeld door Universiteit Maastricht is een computerprogramma welke patiënten begeleidt door een serie van vragen. Door de specifieke antwoorden van de patiënt is het voor de beslishulp mogelijk een behandeling te adviseren die het best aansluit op de wensen en voorkeuren van de patiënt. Op dit moment is de beslishulp nog in ontwikkeling. Om de beslishulp zo bruikbaar mogelijk te maken moet deze nog getest worden door patiënten. Op dit moment is gekozen voor patiënten die al behandeld zijn voor longkanker. Hierdoor wordt het mogelijk de beslishulp aan te passen aan de wensen van patiënten.

Wat is het doel van dit onderzoek?

Evaluatie van besluitvormingsbehoeften van patiënten met longkanker en evaluatie van een prototype van een beslishulp die patiënten met longkanker helpt een keuze te maken voor een behandeling.

Hoe ziet het onderzoek eruit?

Het onderzoek plaatsvinden op een met u afgesproken dag. Uw arts zal u tijdens een controle afspraak vragen of u aan dit onderzoek wil deelnemen, waarna de onderzoeker telefonisch contact met u opneemt. Tijdens dit telefoongesprek zal de onderzoeker deze brief nog eens

met u doornemen en u vragen een toestemmingsverklaring te tekenen. Deze zal u per post of per email worden toegestuurd. Ook zal tijdens dit telefoongesprek een afspraak met u gemaakt worden voor de dag dat het onderzoek zal plaatsvinden. Tijdens de onderzoeksdag zal de onderzoeker u een aantal vragen stellen per telefoon. Vervolgens krijgt u een email met een link naar de beslishulp en vragen wij u deze beslishulp te doorlopen. Wanneer u de beslishulp volledig heeft doorlopen, vragen we u om een vragenlijst in te vullen over de beslishulp. Deze vragenlijst zal u per mail of per post worden toegezonden. In totaal zal het onderzoek ongeveer 2 uur duren.

Is deelname aan deze studie vrijwillig?

Uw medewerking aan deze studie is geheel vrijwillig. U kunt zich op ieder moment, zonder opgave van reden, uit de studie terugtrekken. Dit heeft geen gevolgen voor uw verdere behandeling. Als u stopt met deelname aan dit onderzoek, zullen alle studiegegevens die op u betrekking hebben vernietigd worden.

Kan ik er op vertrouwen dat mijn gegevens vertrouwelijk behandeld worden?

Gegevens over deelname aan en verkregen tijdens deze studie zullen gedurende ten minste 10 jaar bewaard worden. Alleen de onderzoekers en de datamanager van het D-lab, universiteit Maastricht hebben toegang tot de gegevens. Er zullen geen gegevens of resultaten over het onderzoek openbaar gemaakt worden aan derden, behalve in anonieme vorm aan de coördinator van het onderzoek en indien noodzakelijk aan instellingen van de overheid zodat deze kunnen bewaken dat wettelijke bepalingen worden nageleefd. Ook bij publicatie van de resultaten in wetenschappelijke tijdschriften zal de vertrouwelijkheid gehandhaafd blijven.

Wat u verder moet weten

- Er wordt gestreefd om het onderzoek uit te voeren op een dag die voor u goed uitkomt. Voor het onderzoek is het fijn als u het doorlopen van de beslishulp en het invullen van de vragenlijst op dezelfde dag doet als het interview of de dag daarna, maar dit is niet noodzakelijk.

- U heeft het recht om op elk moment alle vragen te stellen over het onderzoek. lndien er tussentijdse informatie is die voor u van belang is zullen wij u dit laten weten.

- lndien u nog vragen heeft of als er bepaalde zaken onduidelijk zijn kunt u contact opnemen met Iva Halilaj, onderzoeker bij Universiteit Maastricht. Zij kan bereikt worden via het centrale telefoonnummer 043 3883549 of via email: i.halilaj@maastrichtuniversity.nl.

3. Informed consent

TOESTEMMINGSVERKLARING

Evaluatie van een beslishulp voor gezamenlijke besluitvorming met betrekking tot behandeling van vroeg stadium niet-kleincellig longkanker.

lk ben over het onderzoek geïnformeerd. lk heb de schriftelijke informatie (versie 1) gelezen. lk ben in de gelegenheid gesteld om vragen over het onderzoek te stellen. lk heb over mijn deelname aan het onderzoek kunnen nadenken. lk heb het recht mijn toestemming op ieder moment in te trekken zonder dat ik daarvoor een reden hoef op te geven. Om mijn toestemming in te trekken moet ik zelf contact opnemen met mijn behandelend arts.

lk stem toe met deelname aan het onderzoek, en geef hierbij tevens toestemming voor het gebruik van mijn medische- en onderzoeksgegevens, zoals omschreven in de informatiebrief (versie 1).

lk verklaar hierbij, dat ik vrijwillig deelneem aan bovengenoemd wetenschappelijk onderzoek.
Naam:

Geboortedatum:

Handtekening:

DatumOndergetekende, verantwoordelijke onderzoeker, verklaart dat de hierboven genoemde persoon zowel schriftelijk als mondeling over het bovenvermelde onderzoek is geïnformeerd. Hij/zij verklaart tevens dat een voortijdige beëindiging van de deelname door bovengenoemde persoon van geen enkele invloed zal zijn op de zorg die hem of haar toekomt.

Naame

Functie

Handtekening: Datum:

N.B. Het origineel van de schriftelijke toestemmingsverklaring dient in het medisch dossier van de patiënt bewaard te worden

4. Interview questions

1. Can you tell me about your lung cancer?

2. Can you tell me about your health now?

3. Can you describe to me which people were involved during your treatment process? What relationship did you have with them?

How does your lung cancer make your daily life changed?

2. What do you miss most of the time before you were treated for lung cancer?

Did you understand all the information you received from a. What would you have liked to know more about your treating doctor? regarding your illness?

Which pieces of information are it a. Did you feel that information was missing?

most important to you (e.g. pros or cons Would you like more technical treatment, duration of treatment, etc.) information or more emotional

Question Sub-questions

What is your name? and why? support? Access to certain information in the hospital?

Do you have any additional a. Why or why not? wanted information? b. What kind of information do you have looked it up and where did you get it looked up?

c. What did you like about this one? information?

4. How would you like more information want to get about the different types treatments? (text, video, etc.)

Were you involved in creating a a. If so, how was this process? treatment choice? b. If not, why don't you have a role played in making a decision?

2. What was the hardest thing about being involved when making a decision?

3. What was the role of your family and friends in the process of making a choice?

4. If you could improve three things in it process of a treatment choice make, what would these be?

5. What Made You Choose for the final treatment?

6. What advice would you give to someone who just been diagnosed with lung cancer?

Are you familiar with using a computer, smartphone or tablet?

Would you like access to a 2. computer program you can use in the process of a

make a treatment choice? Why?

The interview is over, you have helped us a lot with our research. First of all thank you so much for your cooperation. We will now work out the interview. We will send the result to you.

If you do not agree with the details, you can always contact us by email.

Thanks again and have a nice day!

4. METC Approval (translation)

Non- official English translation of Ethical approval

Subject: Non-WMO statement METC 2019-1343 "Pilot study to test a patient decision-making tool for stage 1-11 non-small cell lung cancer"

Dear Ms Halilaj,

Your application for a non-WMO statement* concerning the above-mentioned

research was discussed in the meeting of the Executive Board of the Medical Ethics Review Committee (METC) azM/UM.

In the Executive Board's opinion, your research does not fall within the scope of the Medical Research Involving Human Subjects Act (WMO).

The executive board based its judgement on the "Application form non-MO statement" revision 12396 dated 11-05-2021.

The committee only assessed WMO liability. No further proportionality review of the research took place. Your research group is responsible for carrying out the research correctly according to the applicable laws and regulations. Please draw your attention to this:

- For prospective research, which involves collecting and processing data from subjects, consent from the subjects is required. An example information letter can be found on the CCMO website. It is recommended that the text from paragraph 10 on the processing of data (and possibly body tissue) is also adopted in full in this information letter for research not subject to the WMO.

- For retrospective research, in which data from subjects are collected and processed in coded form, the consent of the subjects is in principle required. The WGBO stipulates the circumstances under which consent may be waived. More information on this can be found on the ELSI service desk website.

- When data is collected from subjects in a study, it should be handled properly as stipulated in the Code of Conduct in Health Research (Code of Good Conduct), General Data Protection Regulation (AVG) and the General Data Protection Regulation Implementation Act (UAVG). The said Code of Conduct can be found on the FEDERA website.

- Whenever in a study bodily material of test subjects is collected and processed, this must be handled correctly as stipulated in the Code of Good Use (Code of Conduct for the Responsible Handling of Bodily Material in Scientific Research), which can be found on the FEDERA website.

- Research using anonymous body material from healthcare is allowed, provided the patient from whom the material originates has not objected. This concerns material that cannot be traced back to the individual in question at all.

- Biobank research requires the consent of subjects. More information on this can be found on the ELSI service desk website.

- Amendments to research should be submitted to the committee via the portal for assessment, so that it can be reassessed whether the research remains non-WMO-compliant.

- Researchers from Maastricht UMC+ must comply with the stipulations of the Maastricht UMC+ research code of conduct.

The committee reminds you that research conducted at Maastricht UMC+ can only be started after receiving an approval decision from the Maastricht UMC+ Board of Governors.

The committee wishes you every success in carrying out your research.
